# Vesicular and Planar Membranes of Archaea Lipids: Unusual Physical Properties and Biomedical Applications

**DOI:** 10.3390/ijms23147616

**Published:** 2022-07-09

**Authors:** Parkson Lee-Gau Chong, Abby Chang, Allyson Yu, Ayna Mammedova

**Affiliations:** Department of Medical Genetics and Molecular Biochemistry, Lewis Katz School of Medicine, Temple University, Philadelphia, PA 19140, USA; abby.chang@temple.edu (A.C.); allyson.yu@temple.edu (A.Y.); ayna.mammedova@temple.edu (A.M.)

**Keywords:** tetraether lipids, diethers, archaea, archaeosomes, drug delivery, controlled release, black lipid membranes, planar membranes, biosensing, coating

## Abstract

Liposomes and planar membranes made of archaea or archaea-like lipids exhibit many unusual physical properties compared to model membranes composed of conventional diester lipids. Here, we review several recent findings in this research area, which include (1) thermosensitive archaeosomes with the capability to drastically change the membrane surface charge, (2) MthK channel’s capability to insert into tightly packed tetraether black lipid membranes and exhibit channel activity with surprisingly high calcium sensitivity, and (3) the intercalation of apolar squalane into the midplane space of diether bilayers to impede proton permeation. We also review the usage of tetraether archaeosomes as nanocarriers of therapeutics and vaccine adjuvants, as well as the biomedical applications of planar archaea lipid membranes. The discussion on archaeosomal therapeutics is focused on partially purified tetraether lipid fractions such as the polar lipid fraction E (PLFE) and glyceryl caldityl tetraether (GCTE), which are the main components of PLFE with the sugar and phosphate removed.

## 1. Vesicular Archaea Lipid Membranes (Archaeosomes)

### 1.1. Formation and Characterization of Archaeosomes

Diethers (also called archaeols) and tetraethers are the dominating lipids in archaea, and their chemical structures and geochemical distributions have been extensively studied (reviewed in [1,2,3,4,5]). The hydrocarbon chains of archaea lipids contain isoprenoid units, may have cyclopentane or cyclohexane rings, and are attached to the *sn*-2 and *sn*-3 positions of glycerol or calditol via ether linkages, whereas the polar headgroup (phosphate or sugar) is at the *sn*-1 position. Archaea tetraether lipids are, in most cases, bipolar macrocyclic compounds containing dibiphytanyl (2 C40) chains. Archaea diether lipids also adopt the *sn*-glycerol-1-phosphate stereochemistry and usually contain diphytanyl (2 C20) chains. Many archaeal lipid analogues have been chemically synthesized, with their physico-chemical properties characterized [6,7,8].

Archaeosomes are defined as liposomes made of, completely or partly, natural ether lipids isolated from archaea or made of synthetic lipids that mimic natural archaea ether lipids. For the sake of discussion, in this article, archaeosomes containing only tetraether or diether lipids are called tetraether or diether archaeosomes, respectively. Archaeosomes containing both archaeal diether and tetraether lipids are referred to as mixed archaeosomes. Archaeosomes containing archaea tetraether (or diether) lipids and non-archaea diester lipids are called hybrid archaeosomes. Liposomes that do not contain archaea or archaea-like lipids are referred to as conventional liposomes. In most cases, tetraether lipids in archaeosomes span the membrane, forming a monolayer structure [9], whereas diether lipids form bilayers. Under certain conditions, tetraether lipids can also adopt a U-shaped configuration and form a bilayer structure [10].

**Figure 1:**
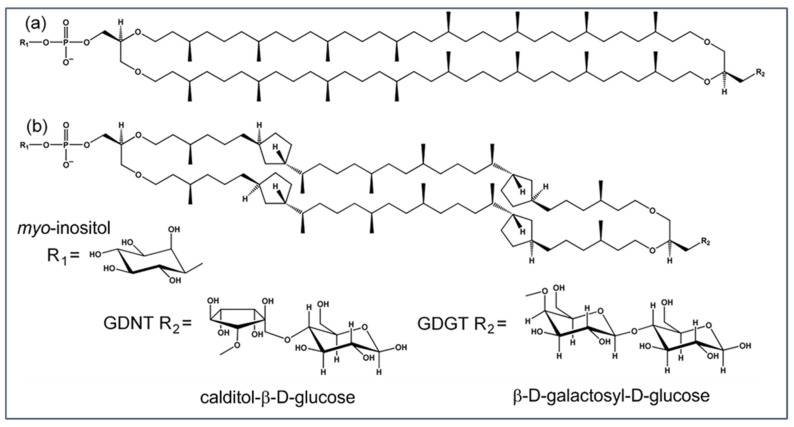
Illustration of structures of tetraether lipids in the polar lipid fraction E (PLFE) extracted from the thermoacidophilic archaeon *S. acidocaldarius.* PLFE contains two components: (**a**) GDGT and (**b**) GDNT. Each has two polar headgroups with one being phospho-*myo*-inositol (R1). The other polar head group is either glycerol-linked β-D-galactosyl-D-glucose (GDGT R2) or calditol-linked β-D-glucose (GDNT R2). Each dibiphytanyl chain may contain 0–8 cyclopentene rings. In this illustration, zero and four cyclopentane rings are present in the dibiphytanyl chain for GDGT and GDNT, respectively. Modified from [16].

**Figure 2:**
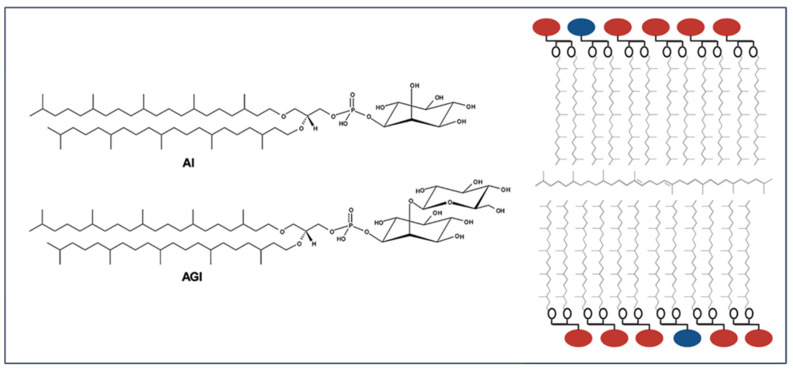
(**Left**) Structures of diether lipids in the polar lipid methanol fraction (PLMF) extracted from the hyperthermophile *Aeropyrum pernix*. (**Right**) Putative membrane structure containing AI (blue circles), AGI (red circles), and the apolar molecule squalane, which resides in the mid-plane space of diether bilayers, similar to the case of squalane in DoPhPC as revealed by neutron diffraction [21,22,23,24,25].

Total lipids (TLs) and total polar lipids (TPLs) extracted from archaea have been used to make archaeosomes for therapeutics delivery [11,12]. TPL archaeosomes may contain both diether and tetraether lipids, and the ratio of diethers to tetraethers varies with archaea type and growth conditions. Compared to TPLs, partially purified archaea ether lipids are compositionally simpler and allow for more straightforward data interpretation. Among the partially purified archaea tetraether lipids, the polar lipid fraction E (PLFE, Figure 1) isolated from the thermoacidophile *S. acidocaldarius* (growth conditions: 75–80 °C and pH 2.5) [13] is most frequently used for archaeosome studies. For the partially purified archaea diether lipids, the polar lipid methanol fraction (PLMF, Figure 2) extracted from the hyperthermophile *Aeropyrum pernix* (growth conditions: 92 °C and pH 7) [14] is of particular interest. To this end, it is important to mention that not all partially purified lipid fractions from archaea can form stable archaeosomes [15]. In this review, we will discuss the unusual physical properties and the biomedical applications of archaea lipid membranes, with special attention focused on the membranes made of PLFE and PLMF.

PLFE contains exclusively tetraether lipids (Figure 1). However, PLFE lipids are still a complex mixture containing two types of hydrophobic cores. The major type (~90%) is glycerol dialkyl calditol tetraether (GDNT, also called calditoglycerocaldarchaeols), in which the dibiphytanyl macrocyclic chain is linked to glycerol at one end and calditol at the other end. The minor component (~10%) of the PLFE hydrophobic core is glycerol dialkyl glycerol tetraether (GDGT, also called caldarchaeols), with the dibiphytanyl chain linked to glycerol at both ends. Each dibiphytanyl chain in PLFE contains up to 8 cyclopentane rings. Three different kinds of polar headgroups have been found on PLFE lipids, namely, phospho-*myo*-inositol, β-D-galactosyl-D-glucose and β-D-glucose [13] (Figure 1). Thus, PLFE lipids are macrocyclic and asymmetric molecules with one polar end (R1, Figure 1) negatively charged at a neutral pH.

Glyceryl caldityl tetraether (GCTE) are the GDNT component of PLFE, with their carbohydrate and phosphate moieties chemically removed [17,18,19]. GCTE (also known as hydrolyzed GDNT (hGDNT) [20]) are still a mixture, with varying numbers of cyclopentane rings in the dibiphytanyl chain.

Lipids in PLMF are exclusively diethers (Figure 2). Specifically, they are C_25,25_-archaeol with two different kinds: 2,3-di-*O*-sesterterpanyl-*sn*-glycerol-1-phospho-1′-(2′-*O*-α-D-glucosyl)-*myo*-inositol (C_25,25_-archaetidyl (glucosyl) inositol; AGI, ~91 mol%) and 2,3-di-*O*-sesterterpanyl-*sn*-glycerol-1-phospho-*myo*-inositol (C_25,25_-archaetidylinositol; AI, ~9 mol%) [14].

Like conventional liposomes, archaeosomes can be prepared as multilamellar and unilamellar vesicles with varying sizes. Unilamellar vesicles can be made by sonication [26], extrusion [27] and the electroformation method [28,29]. The latter makes giant unilamellar vesicles (GUVs, ~10–150 μm) for microscopy studies or as reaction vessels [28,29]. Unilamellarity of tetraether archaeosomes made by the extrusion method was confirmed by the observation of only single rings in the freeze-fracture micrographs [30] and a single layer (~5 nm in thickness) in electron cryotomography [31]. Dark domain images obtained from fluorescence microscopy were used to verify the presence of unilamellarity in GUVs of tetraether archaeosomes [28]. Non-lamellar structures such as cubic phases and hexagonal II phases can occur in tetraether and diether archaeosomes at high temperatures (e.g., 70–85 °C) [32,33]. Surfaces of archaeosomes made of natural archaea lipids carry either neutral or negative charges at a neutral pH, whereas synthetic cationic tetraether-like lipids have been used to make positively charged archaeosomes for gene delivery [34].

### 1.2. Stability and Cytotoxicity of Archaeosomes

#### 1.2.1. In Vitro Stability of Tetraether Archaeosomes

Physical properties of PLFE tetraether archaeosomes have been studied extensively (reviewed in [3,35]) and have been utilized to make archaeosomal therapeutics [16,26,36,37,38,39]. In terms of therapeutics delivery, archaeosomes are favored over conventional liposomes mainly because archaeosomes, particularly those containing tetraether lipids, are of much higher stability in many different aspects.

Membrane volume fluctuations are a critical factor when assessing vesicle stability. Compared to dipalmitoyl-*sn*-glycerol-3-phosphocholine (DPPC, a diester lipid) liposomes, PLFE archaeosomes have relative membrane volume fluctuations 1.6–2.2-times lower at any given temperature over a wide range of temperatures (20–85 °C) [40]. Furthermore, relative volume fluctuations in PLFE archaeosomes are much less temperature-sensitive than those in conventional liposomes [40]. Volume fluctuations of PLFE archaeosomes change only 0.0067% per degree from 30 °C to 80 °C, whereas volume fluctuations of DPPC liposomes change with temperature at the rate of 0.03% per degree below and above the main phase transition temperature and with an even higher rate during the main phase transition [40]. The highly damped volume fluctuations and the low temperature sensitivity indicate that PLFE archaeosomes are extraordinarily stable, even at high temperatures (e.g., 80 °C).

The physical origin of the extraordinary stability of PLFE archaeosomes comes from the unusual chemical structure and the strong inter-molecular interactions of archaea tetraether lipids within the vesicle. Tetraether lipids are stable against autooxidation [41] due to the lack of double bonds in the hydrocarbon chains and the presence of ether rather than ester linkages. The rotational motions of segments in the biphytanyl chains are highly restricted due to the macrocyclic nature of the molecule and the presence of cyclopentane rings and branched methyl groups [42,43]. This rigid hydrophobic trans-membrane structure also leads to strong van der Waals interactions among biphytanyl chains. In addition, there is an extensive hydrogen bond network in the polar headgroup regions [44,45], which also contributes to the great stability of tetraether archaeosomes. The great stability of PLFE-containing archaeosomes also comes from the large negative charges on the vesicle surface [16], which provide strong repulsive forces against vesicle coalescence and aggregation. The remarkable thermal stability of PLFE archaeosomes is manifested in their capability to retain vesicle size and shape, as well as entrap molecules against multiple cycles of autoclaving (~121 °C) [20,46]. The rigid/tight membrane packing in PLFE archaeosomes was also revealed by the completely opposite photoselection effect of the probe 6-dodecanoyl-2-dimethylaminonaphthalene (Laurdan) in PLFE archaeosomes versus conventional liposomes [28]. In conventional liposomes, the chromophore of Laurdan inserts into the bilayer and its excitation dipole moment is in parallel with the membrane normal. In PLFE archaeosomes, due to the tight and rigid membrane packing, Laurdan’s chromophore cannot insert into the membrane core; instead, it resides at the membrane surface with its excitation dipole moment aligned perpendicular to the membrane normal [28].

Membrane volume fluctuations are also tightly associated with solute permeability [47]. Low membrane volume fluctuation explains why the spontaneous leakage rate constant of dyes or drugs entrapped inside tetraether archaeosomes is significantly lower than inside conventional liposomes [27,37,44]. The leakage rate constant of the anti-vasculature drug combretastatin A4 disodium phosphate (CA4P) entrapped in PLFE archaeosomes is two orders of magnitude lower than that in 1-palmitoyl-2-oleoyl-*sn*-glycero-3-phosphocholine (POPC) diester liposomes [37]. Furthermore, due to the packing tightness, entrapment efficiencies in archaeosomes are higher than those in conventional liposomes. As an example, the entrapment efficiency of hypericin in PLFE/DPPC archaeosomes is 1.3–2.5-times higher than that in distearoyl-*sn*-glycerol-3-phosphocholine (DSPC) diester liposomes [26].

Low volume fluctuations also explain why tetraether archaeosomes are stable against vesicle fusion [30,48,49]. The initiation of vesicle fusion requires membrane defects, and the fusion process involves transient non-lamellar membrane structures, both of which do not usually occur in tetraether archaeosomes at ambient conditions.

#### 1.2.2. In Vitro Stability of Diether Archaeosomes

Diether lipids can form bilayer structures, with membrane packing somewhat tighter than diester bilayers due to the less bulky ether linkages compared to the ester linkages. It has been long thought that diether bilayers are not as stable as mono-molecular structures formed by bipolar tetraether lipids. This line of thought has been challenged since the finding that tetraether lipids are absent and diethers are the dominating lipid species in certain hyperthermophilic archaea such as *Aeropyrum pernix* (optimal growth at 90–95 °C, [50]) and *Methanopyrus kandleri* (optimal growth at 100 °C, [51]). Thus, archaeosomes made of PLMF diether lipids (C_25,25_-archaeols, Figure 2) are interesting models of all the hyperthermophilic archaea that have only diether, not tetraether, lipids. PLMF diether archaeosmes do not show any distinct temperature-induced phase transitions and they exhibit strong thermal, pH, and permeability stability [25], similar to that found in PLFE tetraether archaeosomes. Molecular dynamics simulations and small angle X-ray scattering showed that the average area per lipid molecule, the bilayer thickness, and the orientation of the headgroup changes little with temperature [52]. Using a series of PLMF/DPPC hybrid archaeosomes with different molar ratios, it was demonstrated that PLMF diesters made the hybrid archaeosomal membranes more fluid below 50 °C and more tightly packed above 50 °C [53].

Those diether-exclusive extremophiles (e.g., *A pernix*) also contain apolar polyisoprenoids such as lycopanes (major component) and squalenes (minor components). Using synthetic diether lipids, i.e., 1,2-di-*O*-phytanyl-*sn*-glycero-3-phosphocholine (DoPhPC) and 1,2-di-*O*-phytanyl-*sn*-glycero-3-phosphoethanolamine (DoPhPE) and neutron diffraction, as well as other physical techniques, Oger’s group and his collaborators showed that the apolar molecules (squalane) can intercalate into the diether bilayer mid-plane space in parallel with the membrane surface (illustrated in Figure 2), reducing proton permeability, inducing negative membrane curvature, and creating non-lamellar structures at high temperatures and high pressures [22,33]. This unusual membrane structure (right panel, Figure 2) has been proposed as an adaptation strategy of those hyperthermophiles, with only diether lipids, to cope with extremely harsh environments such as high growth temperatures [21,22,33]. At present, it is not clear whether this unusual membrane structure (e.g., right panel, Figure 2) can occur in PLFM archaeosomes made of C_25,25_-archaeols and whether archaea diether bilayers with intercalated apolar isoprenoids are better nano-carriers of therapeutics than those without an apolar isoprenoid.

#### 1.2.3. Other Remarks about In Vitro Archaeosome Stability

When referring to the issue of stability, one needs to consider the vesicle type and lipid composition of archaeosomes. Multilamellar archaeosomes are more stable than unilamellar archaeosomes [54], and tetraether archaeosomes are, in general, more stable than diether archaeosomes. Archaeosomes containing synthetic pegylated tetraether lipids are more stable than pegylated conventional diester liposomes [55]. The bending modulus (i.e., membrane resistance to bending) of GUVs made of GDGT and POPC shows a steady decrease with increasing GDGT till 70 mol%, above which the bending modulus increases sharply with GDGT content [56]. In a similar hybrid archaeosome system, i.e., PLFE/DPPC, zeta potential changes with PLFE content also in a biphasic manner, showing the most negative value (i.e., most resistant to vesicle coalescence) at 60 mol% PLFE [16].

It is also necessary to know the exact experimental conditions when archaeosome stability is monitored. For example, the sucrose leakage rate from archaeosomes made of total lipids isolated from *T. acidophilum* can vary from 10% at pH 2.5 to 80% at pH 1.5 within 90 min at 37 °C [54]. Therefore, while it is true that archaeosomes are, in general, more stable against acidic conditions, we need to be reminded that archaeosome stability can in fact decrease significantly as pH decreases. As another example, PLFE archaeosomes can sustain at least six cycles of autoclaving, while conventional diester liposomes made of 1-palmitoyl-2-oleoyl-*sn*-phosphocholine (POPC) cannot hold the size and shape with just one cycle of autoclaving [20]. This result led to the conclusion that tetraether archaeosomes are more stable against autoclaving than conventional liposomes. While this general conclusion is correct, it is important to note that PLFE archaeosomes are stable against autoclaving only for pH 4–10. At pH < 4, the size of PLFE archaeosomes increases with autoclaving cycles indicating vesicle aggregation or membrane disruption occurring at extreme low pHs [20].

Like cholesterol, PLFE and GCTE can stabilize liposomal membranes [19,37]. To evaluate whether PLFE or cholesterol is a better liposome stabilizing agent, a parallel comparison was made by using the same drug (CA4P) leakage and cytotoxicity (CyQuan) assays and it was found that the CA4P leakage rate constant in 20 mol% PLFE/80 mol% POPC [37] is significantly smaller than that in 20 mol% cholesterol/80 mol% POPC [57]. Apparently, PLFE is more efficient than cholesterol in keeping liposomal drugs.

As a stabilizing agent for liposomal drugs, PLFE tetraether lipids have advantages over cholesterol [37]. First, the amount of cholesterol in a liposome is restricted by the cholesterol solubility limit, which is 66.7 mol% for phosphatidylcholine liposomes [58]. In contrast, liposomes can be formed with 0–100% PLFE. Second, like cholesterol, PLFE increases membrane order. However, the magnitude of the membrane order increase that can be changed by PLFE in PLFE/POPC hybrid systems is greater than that changed by cholesterol in cholesterol/POPC liposomes. Therefore, in terms of tightening membrane packing in the hydrophobic core, PLFE can provide a wider range of alterations than cholesterol. Third, although cholesterol can tighten membrane packing, cholesterol does not change vesicle zeta potential and, thus, does not increase liposome stability against colloidal aggregation/coalescence. In sharp contrast, the zeta potential of PLFE/POPC liposomes can be changed from slightly negative to much more negative [37]. When the zeta potential becomes <−30 mV, the colloidal particles are usually considered as stable against particle aggregation and coalescence during long-term storage. Fourth, in the case of drug leakage from cholesterol/POPC liposomes [57], the leakage rate does not change with cholesterol mole fraction in a monotonic manner; instead, the drug leakage rate changes with cholesterol in an alternating manner following the principle of sterol superlattice formation [59]. Finally, it is important to note that PLFE archaeal lipids, unlike cholesterol, are chemically inert and not linked to any human disorders.

#### 1.2.4. Archaeosome Cytotoxicity

The in vitro cytotoxicity study of archaeosomes made of lipids isolated from the hyperthermophilic archaeon *Aeropyrum pernix* K1 yielded mixed results [60]. *A. pernix* archaeosomes were nontoxic to human liver hepatocellular carcinoma HepG2 and human epithelial colorectal adenocarcinoma CACO-2 cells [60]. However, these archaeosomes exhibited mild toxicity to Chinese hamster ovary and mouse melanoma B16-F1 cells and showed a strong cytotoxic effect on human endothelial umbilical vein cells EA.hy926 [60]. Hybrid archaeosomes made of 10 mol% PLFE and 90 mol% DPPC with entrapped photosensitizers showed negligible hemolytic toxicity and only a slight increase (5–12 s) in blood coagulation time [26].

Since approximately 25–30% of the population has methanogens in the gastrointestinal (GI) tract, oral cavity, and skin, and since methanogens contain both diether and tetraether lipids, it has been suggested that archaeal lipids and archaeosomes are safe to humans [18]. Indeed, archaeosomes from TPLs of *Halobacterium salinarum*, *Methanobrevibacter smithii and Thermoplasma acidophilum* were found non-toxic in mice with no adverse effects such as swelling, redness, granuloma, or abscess formation at injection sites [61,62,63]. However, at the high intravenous dose of 140 mg/kg/day, archaeosomes made of TPLs of the halophiles, *Halobacterium salinarum* and *Natronobacterium magadii*, showed signs of toxicity such as drop in body temperature, loss in body weight, and enlarged spleens [64]. Apparently, more in vivo toxicity studies on archaeosomes are needed. Urine analyses suggest renal excretion is involved in the metabolism of tetraether lipids in rats [18]. However, exactly how archaea lipids are metabolized and cleared from animals remains elusive.

Nevertheless, most studies seem to show that archaeosomes, when used not in exceedingly high doses, have no or little cytotoxicity, which, in conjunction with great vesicle stability, would make archaeosomes superior alternatives to conventional liposomes for biomedical applications. Thus, archaeosomes can be potentially useful as nano-carriers of therapeutics via different administration routes such as intravenous injection, nasal spray, or oral intake. In the next two sections, we shall review the use of archaeosomes as nano-carriers of therapeutics and as vaccine adjuvants.

### 1.3. Archaeosomes as Nanocarriers of Therapeutics

#### 1.3.1. Tetraether Archaeosomes for Oral Delivery

Tetraether archaeosomes are likely to be excellent nano-carriers for oral delivery (reviewed in [65]) because tetraether lipids are stable in acidic pH, and proton permeability across tetraether archaeosomal membrane is much lower than that in liposomes composed of diester lipids [27,44,54]. In addition, tetraether archaeosomal peptides have increased oral availability [18] and are stable against digestive enzymes in the GI tract [41,62], bile salts in the intestinal lumen [66], and acidic environments in GI fluid [67] (Table 1). PLFE archaeosomes can keep entrapped insulin in mice for a longer time after oral administration compared to conventional liposomal insulin [67] (Table 1). The digestive enzymes (such as phospholipase A2, pancreatic lipase, phosphoinositol specific phospholipase C, and phospholipase B) prefer to act on ester over ether substrates. In general, hydrolytic enzyme activity is decreased in this order: conventional liposomes > diether archaeosomes > tetraether archaeosomes [41]. In addition to achieving higher stability in acidic and digestive environments, glyceryl caldityl tetraether (GCTE) archaeosomes coated with cell-penetrating peptides (CPP) can overcome the problem of low mucosal permeation, as evidenced by more effective oral delivery of vancomycin into the liver in mice than naked GCTE archaeosomes [68] (Table 1).

#### 1.3.2. Tetraether Archaeosomes for Intravenous Delivery

Intravenous delivery of tetraether archaeosomal therapeutics looks promising in the sense that tetraether archaeosomes are stable against serum proteins and blood hydrolytic enzymes such as phospholipase A2 [41]. In this regard, diether archaeosomes are inferior [41].

Conventional liposomes in the circulation can be subject to opsonization by certain serum proteins such as immunoglobulins and fibrinogen, which facilitate liposome uptake by a wide variety of phagocytic cells called the reticuloendothelial system (RES). The more hydrophobic the liposome surface is, the more likely it is removed by RES.

Many tetraether archaeosomes are negatively charged at a neutral pH. The stability of negatively charged conventional liposomes in the circulation is dependent on liposome composition [72]. Anionic liposomes without sugar moieties on the surface can be subject to opsonization by serum proteins and subsequently removed by RES [73]. However, negatively charged liposomes coated with gangliosides (a glycosphingolipid with one or more sialic acid) have been shown to be stable in the serum and have long circulation times [74,75]. Like gangliosides, PLFE lipids are negatively charged and have multiple sugar moieties in the polar head groups, which should be able to form a hydrophilic layer on the archaeosome surface and, consequently, help evade detection by RES and possess long circulation times.

Interestingly, Plenagl et al. reported that DPPC/PLFE archaeosomes (molar ratio = 9:1) absorb more serum proteins than DSPC ester liposomes [26]. However, it is not known if the absorbed proteins are opsonins or dysopsonins, which would decrease or increase the circulation time, respectively. Future work should put more effort into determining the in vivo circulation times of tetraether archaeosomes and the archaeosome proteome (or archaeosome corona) [76]. Nevertheless, it is of interest to mention that the in vitro uptake of anionic mixed archaeosomes derived from methanogens (with diethers as the major lipid component) by macrophages was 3–50 times greater than that of anionic conventional liposomes [62].

### 1.4. Archaeosomes That Can Conduct Controlled Release and Target Delivery

One of the main challenges of using archaeosomes for therapeutics delivery has been the establishment of an efficient method to trigger the release of entrapped content from the otherwise extremely stable structure. Discussed below are the approaches that have been used to deal with this problem (Table 1).

#### 1.4.1. Archaeosomal Photosensitizer

Light irradiation on tumors and their microvasculature structures can generate reactive oxygen species (ROS) from the photosensitizers (e.g., hypericin and protoporphyrin IX) embedded in hybrid archaeosomes [26,38,69,70,77]. The resulting ROS cause oxidative damage to tumor cells and their microvasculature structures, and the high stability of archaeosomes makes this photodynamic event more efficient. Archaeosomal hypericin, for example, had enhanced entrapment efficiency and created approximately twice as much cytotoxicity through photodynamic disruption than conventional liposomal hypericin on SK-OV-3 cells and the chicken chorioallantoic membrane (CAM) angiogenesis model, with negligible hemolytic toxicity, and only a small increase in coagulation time [26]. Similar results were obtained with other photosensitizers [38,69,70,77]. The mole fraction of tetraether lipids in the archaeosome formulation is important. Archaeosomal protoporphyrin IX with 62 mol% PLFE and 38 mol% DPPC produced localized suppression of angiogenesis in the irradiated area without quiescent vasculature damage and massive thrombosis, whereas archaeosomes with 9 mol% PLFE generated more disruptive side effects, which is less desirable [38,69].

#### 1.4.2. Thermosensitive Archaeosomes

Several thermosensitive conventional liposomes (TSLs) [78,79,80] have been formulated to attain significant drug release in response to local mild hyperthermia treatment (i.e., raising the local body temperature from 37 °C to 42–44 °C) by virtue of the phase transition of DPPC at 41.5 °C, achieving targeted delivery and reducing adverse effects of liposomal drugs. However, stability has been the issue of the conventional thermosensitive liposomes. First, they are made of diester lipids, which are susceptible to attacks from serum proteins, enzymes, and hydrolysis/oxidation reactions. Second, most of them contain cholesterol as the stabilizing agent. Cholesterol is of concern as it can be oxidized and excessive oxidized cholesterol in the body leads to atherosclerosis, and atherosclerosis and cancer share common molecular pathways of disease development and progression [81]. Furthermore, cholesterol stabilizes gel phase and resists thermal shock [82,83]. Third, many TSLs, including ThermoDox (a TSL currently under clinical trials), contain PEGylated lipids. PEGylation provides long circulation times [83,84]. However, PEGylated lipids reduce drug loading, leading to decreased drug exposure at the tumor site [85,86,87]. Moreover, the degradation of pegylated lipids can cause an increase in membrane permeability [88], and PEGylated lipids may elicit the generation of a PEG-specific antibody that causes accelerated blood clearance [89].

**Figure 3:**
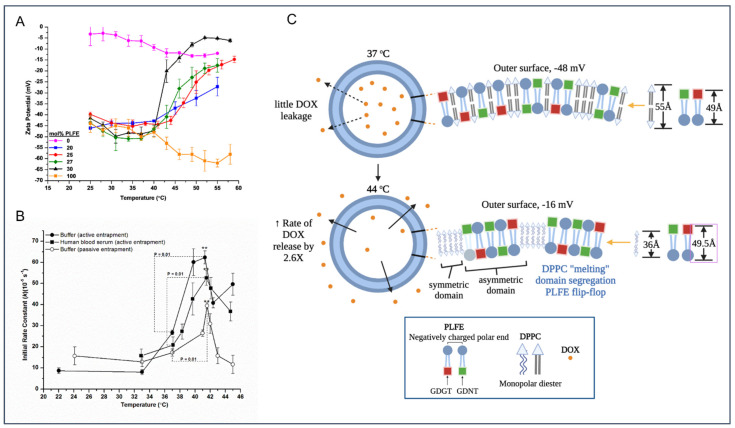
Thermosensitive archaeosomes made of PLFE and DPPC (molar ratio 3:7) exhibit unusual physical properties. (**A**) zeta potential of this formulation undergoes a dramatic change from very negative at and below the body temperature to much less negative at 44 °C. (**B**) In conjunction with this temperature jump and zeta potential change, there is an abrupt increase of the rate of drug release from the archaeosomes. (**C**) The plausible explanations of the zeta potential change involve major archaeosomal structural changes such as DPPC melting, PLFE flip-flop, domain segregation due to hydrophobic mismatch [90], polar headgroup exposure [91], etc. (**A**,**B**) are taken from [16] with permission.

Hybrid archaeosomes containing PLFE and DPPC can be tailored to be thermosensitive, which not only circumvent the problems associated with cholesterol and pegylated lipids, but also display novel physical properties as described below [16] (Figure 3). Specifically, when using archaeosomes made of PLFE and DPPC at the molar ratio 3:7, the anticancer drug doxorubicin (DOX) was readily incorporated into the archaeosomes and the extraordinary stability of archaeosomes was retained due to both tight/rigid membrane packing and large negative charges on the archaeosome surface (Figure 3). As a result, a few entrapped DOX molecules leaked out and no vesicle coalescence occurred at ≤37 °C [16]. Once the temperature jumped from 37 °C to 42–44 °C (mimicking hyperthermia treatment), the entrapped DOX molecules underwent an abrupt release (2.6 times faster) (Figure 3B), resulting in more cell death [16]. It was proposed that the increased drug release and cell death engendered by this small temperature jump was due to major structural changes in the archaeosomes, probably involving DPPC melting, PLFE transmembrane flip-flop, conformational changes in PLFE polar headgroups, domain segregation, and a reduction in particle surface charges [16] (Figure 3C). These structural changes were proposed largely based on the observation that the zeta potential of PLFE/DPPC (3:7) archaeosome was abruptly changed from very negative (−48 mV) to much less negative (−16 mV) when the temperature was raised from 37 °C to 42–44 °C [16] (Figure 3A).

Such a large temperature-induced zeta potential change has not been seen in other liposome systems, and this could be a unique physical property for hybrid archaeosomes containing asymmetric macrocyclic tetraether lipids with one polar end negatively charged (e.g., PLFE). This unusual physical property of hybrid archaeosomes has appealing application implications because surface charge (as reflected by the zeta potential value) can affect the interactions of liposomes with cells in the surroundings. Being able to alter the surface charge would make the liposomes more versatile and able to find more applications. For example, it has been reported that slightly negatively charged (−15 mV) liposomes have better in vivo circulation time and inflamed joint targeting than highly negatively charged (−30 mV) liposomes for rheumatoid arthritis treatment [92].

In short, mixtures of PLFE/DPPC (3:7) are smart thermosensitive archaeosomes, which are extraordinarily stable at body temperature, yet they are thermosensitive, which makes them capable of conducting controlled release and altering their interactions with target cells through surface charge changes upon mild temperature elevations [16].

### 1.5. Archaeosomes as Vaccine Adjuvants

#### 1.5.1. Conventional Liposomal Vaccines

Subunit vaccines have become increasingly popular in vaccine development due to their excellent safety profiles. A prominent example is the COVID-19 vaccines from Pfizer and Moderna, which exploit mRNA for a particular virus protein, i.e., the spike protein. However, the use of pathogen subunits as the antigen is less immunogenic than the use of live pathogens or killed pathogens; thus, subunit vaccines require adjuvants to boost antibody production.

Conventional liposomes made of diester lipids and cholesterol have been used for as long as adjuvants in vaccines and some of them have been approved for human use (reviewed in [93,94,95,96]). Animal and clinical studies showed that antigens entrapped in liposomes elicit more antibodies and less antibody-mediated allergic reactions than free antigens [97]. A notable liposome-based vaccine adjuvant system, AS01, has been studied in multiple clinical trials for different diseases and was FDA approved as a shingles vaccine (Shingrix) in 2017 [98].

When used as vaccine adjuvants, liposomes can carry not only antigens (e.g., peptides and proteins) but also immunostimulators. The adjuvant property of liposome vaccines comes from their interactions with antigen presenting cells (APCs) and their ability to enhance the uptake of antigens and immunostimulators by APCs [94], as a result, both innate and adaptive immune responses are enhanced.

Cationic liposome vaccines are more immunogenic, taken up by APCs more readily and produce more antibodies than anionic liposome vaccines [94]. In addition, liposomes with their membranes more tightly packed induce more cell-mediated immune responses due to increased adjuvant stability [99], and PEGylation on liposome surface can affect the immune responses such as the production of cytokines [100]. The administration route, particle size, and methods of loading antigens and immunostimulators are also factors influencing the immune responses of liposome vaccines [94].

#### 1.5.2. Archaeosomal Vaccines

Archaeosomes have been used as vaccine adjuvants and have shown several advantages over conventional liposomes [63,101,102]. Archaeosomes have high thermal and pH stability and enhanced immunostimulatory effects. They can be taken up by macrophages via phagocytosis with higher efficiency than conventional diester liposomes [103]. Like conventional liposomes, archaeosomes exhibit highly effective adjuvant activities in animal models and induce strong humoral (antibody) and cell-mediated (T-cells) immune responses and protective immunity when used to carry antigens of various kinds. Early applications of archaeosomes as vaccine adjuvants focused on the use of total polar lipid extracts (TPL) from the archaea. The drawback of this approach is the chemical heterogeneity of natural archaea lipids. As an example, the TPL from *Methanobrevibacter smithii* contains ~40–50% tetraethers and ~50–60% diethers. More recent studies on archaeosome vaccine adjuvants emphasize the use of chemically pure, semi-synthetic archaeal lipids such as sulfated lactosylarchaeol (SLA) [104,105,106,107]. TPL-based archaeosomes are taken up by bone marrow-derived macrophages and dendritic cells, whereas SLA-based archaeosomes target preferentially bone marrow-derived macrophages. The reason for the difference is the lack of phosphoserine (PS) in SLA-based archaeosomes, while TPL archaeosomes have lipids with PS as the headgroup, which can bind PS receptors on dendritic cells.

Antigens (e.g., ovalbumin antigen) can be encapsulated into or admixed with SLA archaeosomes [104]. Admixed archaeosomes are prepared by adding the antigen to the pre-formed archaeosomes. with the desired antigen-to-lipid ratio. Compared to encapsulated archaeosomes, admixed archaeosomes are easier to prepare, provide a more consistent antigen-to-lipid molar ratio, and lead to much less antigen loss during archaeosome preparation. In addition, admixed SLA archaeosomes (e.g., 1 mg SLA and 10–20 mg ovalbumin per vaccine dose [108]) can induce stronger in vivo immunogenicity and higher efficacy in mice than encapsulated SLA archaeosomes [104].

Alum, aluminum containing adjuvants, is commonly used in vaccines for hepatitis A and B; however, has struggled to induce strong IgG2a and cell-mediated immunity, particularly in T1 cells. Archaeosomes have been found to strongly induce both IgG1 and IgG2a34, as well as T1 and T2 cells in splenocytes, relative to the Alum group. In addition, archaeosome adjuvants had superior activity over the alum adjuvants compared to the cytokine secretion of splenocytes of vaccinated mice in test groups with negative control groups [109].

Different archaeosome adjuvant formulations may also affect the outcome of immune responses. Stark et al. [107] evaluated different archaeosome formulations as an adjuvant to the H1N1 influenza hemagglutinin (HA) protein and compared immune responses (anti-HA IgG and hemagglutination inhibition assay titers) as well as protection to an influenza A virus. It was found in a murine model that an admixed archaeosome formulation composed of SLA can give equal or better protection compared to a squalene-based oil-in-water nano-emulsion, AddaVax™ or the traditional antigen-encapsulated archaeosome formulations. SLA-admixed archaeosomes can induce potent anti-human influenza hemagglutinin in young, aged, and pregnant mice. This viral protection can also be passed down to a pregnant mother’s offspring [107].

## 2. Archaeal Planar Membranes

While vesicular archaea lipid membranes (archaeosomes) are promising vaccine adjuvants and nano-carriers of therapeutics, as reviewed above, planar archaea lipid membranes, either free-standing over pinholes/nanopores or solid supported, also find their potential applications as durable platforms of biosensors and novel coatings on biomedical devices.

### 2.1. Free-Standing Archaea Lipid Planar Membranes

#### 2.1.1. Stability

Free-standing planar lipid membranes (also called black lipid membranes, BLM) over micro- or nano-pores in a solid thin film are excellent platforms for biophysical studies and biosensing applications [110]. However, instability of the BLM made of conventional diester lipids has been a serious drawback [111]. Archaea tetraether lipids have been used to make BLMs (reviewed in [112,113]). Earlier studies [114,115,116] used archaea lipid extract to make free-standing planar membranes over micro-pores in a Teflon partition; however, those membranes lasted only 5–6 h or less, which was not superior to BLM made from diester lipids. The electric breakdown was attributed to the formation of hydrophilic pores in the membrane [116]. In contrast, in a recent study, PLFE has been shown to form stable BLM on the micro-pores of polydimethylsiloxane (PDMS) thin films embedded in a printed circuit board-based fluidics or in a glass/silicon microchip, exhibiting a constant electrical impedance for at least 55 h at 11–39 °C [117,118]. In a more recent study, it was demonstrated that BLM_PLFE_ formed over a pinhole on a cellulose acetate partition in a dual-chamber Teflon device exhibited even more remarkable stability, showing a constant capacitance (~28 pF) for at least 11 days [113]. The extended stability may be related to the substrate material. A cellulose acetate partition has more groups capable of forming hydrogen bonds than PDMS. It appears that the chemical structure of the lipid used to make the BLM and the substrate used to hold the BLM both are critical for making stable BLM. In short, the remarkable stability of BLM_PLFE_ on a cellulose acetate partition has been attributed to strong PLFE-PLFE and PLFE-substrate interactions [113].

Not all archaea tetraether lipids can form stable BLM. For tetraether lipids isolated from the thermoacidophilic archaeon *Sulfolobus solfataricus*, the GDNT, not GDGT, component was able to form stable BLM [114]. While BLMs made of PLFE (with GDNT as the major component) can last for ≥11 days, BLMs made of tetraether lipids isolated from microvesicles (with GDGT as the major component) released from the same archaea cell (*S. acidocaldarius*) can last only for 8 h before membrane dielectric breakdown [113]. Additionally, pre-treatment of the torus with lipids and the use of a particular organic solvent to make lipid stock solutions also appear to be critical for the formation of stable BLM.

#### 2.1.2. Possible Applications

**Figure 4:**
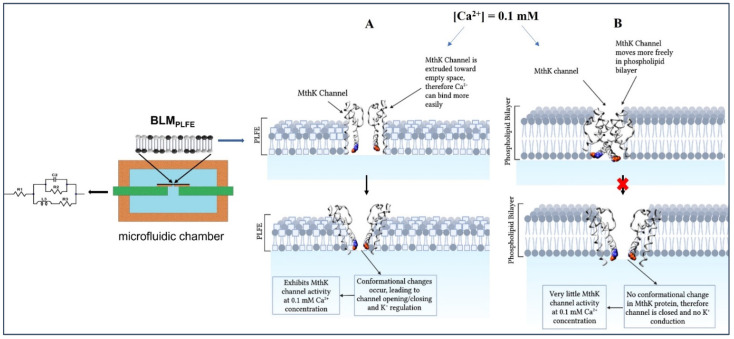
Proposed Mthk behaviors in (**A**) BLM_PLFE_ versus (**B**) BLM_diester_ in a microfluidic chamber based on the findings shown in [113]. The blue and red protein structures represent the positions of Glu-92 and Glu-96, respectively, within the MthK channel [122].

Small peptides or ionophores, such as nonactin, valinomycin, and gramicidin, can insert into BLM_BTL_ and exhibit membrane conductance [114,115]. A recent study showed that despite its large molecular weight, membrane-bound protein MthK, a Ca^2+^-gated K channel (~220 KDa), can not only insert into BLM_PLFE_ and exhibit channel activity, but also show channel opening/closing at calcium concentrations as low as 0.1 mM [113]. At such a low calcium concentration, no MthK channel activity is detectable in diester lipid membranes. For example, Mthk did not show channel activities in BLM made of 1-palmitoyl-2-oleoyl-*sn*-glycero-3-phospho ethanolamine and 1-palmitoyl-2-oleoyl-*sn*-glycero-3-phosphoglycerol (molar ratio 3/1) at [Ca^2+^] = 0.1–1.0 mM at pH 7.6 [119]. This unusually high sensitivity of MthK to calcium in BLM_PLFE_ has been explained in terms of strong PLFE-PLFE (thus tight membrane packing) and MthK-PLFE interactions [113]. As a result, MthK’s topography in BLM_PLFE_ is most likely to be significantly different from that in BLM_diester_. It was proposed (Figure 4) that when embedded in archaea tetraether lipid membranes, MthK is more extruded toward the space outside the membrane and the “rocking” motion [120] of the channel protein in the membrane is more damped, as is the case of bacteriochlorophyll *a* (BChl*a*) in the light-harvesting polypeptide (LH)/BChl*a* complex, which was found to be more homogeneously and perpendicularly oriented with respect to the membrane surface in archaea tetraether lipid membranes than in diester lipid membranes [121]. This protein topography could lead to stronger binding of Ca^2+^ to the MthK RCK domains, which triggers a series of protein conformational changes, leading to channel opening and K^+^ conduction (Figure 4) [113]. MthK in BLM_PLFE_ may be developed into robust microchips to detect toxins. The fabrication of the prototype of such microchips has been previously described [117,118].

### 2.2. Solid-Supported Planar Membranes

Phospholipid bilayers supported by a solid substrate are thought to be more stable and robust compared to black lipid membranes made of conventional diester lipids. Recent progress on BTL_PLFE_ (discussed above) may render this statement incorrect. In these planar membranes, fluidity is maintained by a thin layer of trapped water (2–10 Å) between the substrate and the bilayer, allowing for more surface-specific analytical techniques to be utilized [123].

To date, the applications of solid-supported planar membranes made of archaea or archaea-like lipids are largely limited to biophysical and electrophysiology studies. The physical tools used for these studies include Langmuir-Blodgett films, atomic force microscopy, tensiometry, ellipsometry, small angle X-ray scattering and fluorescence microscopy. The major findings include (1) the stereochemistry of a central cyclopentane ring is critical for controlling the conformation of the corresponding bipolar tetraether lipids [124,125]; (2) archaea tetraether lipids in solid-supported planar membranes can adopt a U-shaped (thickness 1.5–1.8 nm) or a stretched (4–5 nm) conformation [126,127]; (3) domain formation occurs in the mixture of tetraether and diester lipids [89,128]; (4) tetraether lipid monolayers built on the S-layer protein support are more stable (lasting for two days) than those without the S-layer support [129,130]; and (5) squalene, a major non-polar membrane lipid pigment found in archaea (discussed in Section 1.1), has a role in modulating membrane packing and lateral organization [131]. How to engineer solid-supported archaea lipid membranes for biosensing technology has not yet been materialized.

### 2.3. Archaea Lipid Planar Membranes as Novel Coating Materials on Biomedical Devices

Due to the high stability against various environmental stressors and the bipolar nature of the chemical structure, archaeal tetraether lipids are attractive coating materials to modify the surfaces of medical devices and implants to prevent or reduce biofilm formation and bacterial infections. An immobilization matrix was developed from monolayers formed by bipolar tetraether lipids isolated from the archaeon *Thermoplasma acidophilum* and was used on the surface of cellulose dialysis membranes [132] and medical silicon elastomers [133]. Specifically, the substrate silicone rubber was hydrolyzed by sodium hydroxide and then aminosilanized by aminopropyl trimethoxysilane to create amino groups on the substrate surface. The sugar moieties (mainly gulose, [134]) on *T. acidophilum* tetraether lipids, on the other hand, were removed to yield the bare hydrophobic core, which were then reacted with cyanuric chloride at both polar headgroups. When cyanuric chloride-activated caldarchaeols in aqueous solution are spread to the solid support [135], the cyanuric chloride at one of the two polar lipid headgroups can react with the amino groups on the substrate, followed by self-assembly to form planar lipid membranes on the substrate surface. The cyanuric acid at the other polar end of tetraether lipids can be further chemically modified to meet various application needs [133]. Such archaea lipid-coated silicone has been tested in vitro and in vivo, showing no significant evidence for cytotoxicity, genotoxicity, sensitization, irritation, blood incompatibility, allergenic and mutagenic reactions. Additionally, it has been shown that such coatings on silicon rubbers are sterilizable and antiadhesive for bacteria growth, a property that can be used as a coating for tubular catheters to fight against peritoneal dialysis infection [133]. The bivalent linker cyanuric chloride was also used to modify the surface of cellulose dialysis membranes, which reduced the attachment of serum proteins and bacteria on the surface [132]. However, to date, there are no detailed reports on whether and to what extent tetraether lipids are better antiadhesive or antimicrobial coating materials on solid surfaces compared to polymers such as polyethlene glycol (PEG), which can also generate an extensive hydrogen-bonding hydration layer on the substrate surface, hindering bacterial growth.

## 3. Summary

Despite the fact that many efforts have been devoted to studying archaea lipid membranes and finding their applications in the past four decades, the unusual and interesting physical properties of membranes containing archaea or archaea-like lipids have been reported in recent years. In this article, we have particularly reviewed the works of apolar squalanes in diether bilayers (Figure 2), thermosensitive archaeosomes (Figure 3), and MthK in BLM_PLFE_ (Figure 4). These findings point to a couple of future research directions: (i) exploring the biophysical details of membrane protein behaviors in archaea tetraether lipid environments and (ii) studying membrane lateral organization and dynamics in hybrid archaeosomes or planar membranes (e.g., tetraether/diester mixtures, such as PLFE/DPPC). Additional novel physical properties of archaea lipid membranes, plus their great stability and the non-toxicity, could lead to many more biomedical applications.

## Figures and Tables

**Table 1:** Main features of recent PLFE and GCTE archaeosomal therapeutics.

Formulation (Molar Ratio)	Prepared by	Therapeutics Entrapped	Cultured Cells	In Vivo Model	Controlled Release by	Major Findings	References
PLFE/DPPC (1:9)	sonication	hypericin (photosensitizer)	human ovarian carcinoma cells (SKOV-3)	CAM	light irradiation	hypericin archaeosomes are suited for antivascular targeting	[26]
PLFE/DPPC (9:91), (29:71), and (62:38)	sonication and extrusion	protoporphyrin IX (photosensitizer)	SKOV-3 and mouse fibroblast L929 cells; CAM	CAM	light irradiation	PLFE-stabilized PDT liposomes suppressed angiogenesis and removed thrombosis in the chick	[38,69]
PLFE/DSPC (10:90)	sonication and extrusion	curcumin (photosensitizer)	SKOV-3 and primary human coronary artery endothelial cells (PCS-100-020™ cells)	CAM	light irradiation	Archaeosomes are hemocompatble, coagulation time < 50 s	[70]
PLFE	sonication	insulin	Caco-2 cells	mice (oral)	N/A	Archaeosomal insulin leads to lower blood glucose than conventional liposomal insulin	[67]
PLFE/DPPC (3:7)	extrusion	doxorubicin (Anticancer drug)	human breast cancer MCF-7 cells	N/A	hyperthermia-like treatment	an abrupt DOX release and a dramatic change in particle surface charge	[16]
GCTE/ cholesterol/EPC (5:10:85), with and without CPP	speed mixing with glass beads	vancomycin (antibiotic)	Caco-2 cells	mice (oral)	N/A	GCTE and CPP work synergistically in enhancing oral bioavailability and anti-infection efficacy	[68,71]
GCTE/DPPC (3:1), GCTE/EPC (4:1), other molar ratios	extrusion	octreotide (peptide inhibitor of exocrine and endocrine secretion)	N/A	rats (oral)	N/A	a 4.1-fold increase in oral bioavailability	[18]
GCTE/cholesterol/EPC (5:10:85)	speed mixing with glass beads	myrcludex B (Hepatitis B entry inhibitor)	N/A	rats (oral)	N/A	7% of the initial dose detected in the liver (a 3.5-fold increase vs. control)	[17]

## Data Availability

Not applicable.

## Abbreviations

AGI	2:3-di-*O*-sesterterpanyl-*sn*-glycerol-1-phospho-1’-(2’-*O*-α-D-glucosyl)-*myo*-inositol (C_25,25_-archaetidyl (glucosyl) inositol)
AI	2,3-di-*O*-sesterterpanyl-*sn*-glycerol-1-phospho-*myo*-inositol (C_25,25_-archaetidylinositol)
APC	antigen presenting cells
BLM	black lipid membranes
CAM	chicken chorioallantoic membrane
CA4P	combretastatin A4 disodium phosphate
CPP	cell penetrating peptide
DoPhPC	1,2-di-*O*-phytanyl-*sn*-glycero-3- phosphocholine
DoPhPE	1,2-di-*O*-phytanyl-*sn*-glycero-3-phosphoethanolamine
DOTAP	1,2-dioleoyl-3-trimethylammonium-propane
DOX	doxorubicin
DPPC	dipalmitoyl-*sn*-glycerol-3-phosphocholine
DSPC	distearoyl-*sn*-glycerol-3-phosphocholine
EPC	egg phosphatidylcholine
GCTE	glyceryl caldityl tetraether
GDGT	glycerol dialky glycerol tetraether (caldarchaeols)
GDNT	glycerol dialky calditol tetraether (calditolglycerocaldarchaeol)
GTGT	glycerol trialkyl glycerol tetraether
GUV	giant unilamellar vesicles
hGDNT	hydrolysed glycerol-dialkyl-nonitol tetraether
Laurdan	6-dodecanoyl-2-dimethylaminonaphthalene
PDMS	polydimethylsiloxane
PLMF	the polar lipid methanol fraction
PLFE	the polar lipid fraction E
POPC	1-palmitoyl-2-oleoyl-*sn*-glycero-3-phosphocholine
PS	phosphoserine
RES	the reticuloendothelial system
SLA	sulfated lactosylarchaeol
TPL	total polar lipids
TL	total lipids
TSL	thermosensitive liposomes
